# A Randomized, Double‐Blind, Placebo‐ and Positive‐Controlled, 4‐Period Crossover Study of the Effects of Solriamfetol on QTcF Intervals in Healthy Participants

**DOI:** 10.1002/cpdd.867

**Published:** 2020-09-15

**Authors:** Katie Zomorodi, Dan Chen, Lawrence Lee, Dennis Swearingen, Lawrence P. Carter

**Affiliations:** ^1^ Jazz Pharmaceuticals Palo Alto California USA; ^2^ Celerion Tempe Arizona USA

**Keywords:** ECG, JZP‐110, pharmacokinetics, QTc, safety, Sunosi, solriamfetol

## Abstract

Solriamfetol, a dopamine and norepinephrine reuptake inhibitor, is approved (United States and European Union; Sunosi) to treat excessive daytime sleepiness associated with narcolepsy (75‐150 mg/day) or obstructive sleep apnea (37.5‐150 mg/day). A thorough QT/QTc study assessed solriamfetol effects on QT interval (Fridericia correction for heart rate; QTcF). This randomized, double‐blind, placebo‐ and positive‐controlled, 4‐period crossover study compared single doses of 300 and 900 mg solriamfetol, 400 mg moxifloxacin, and placebo in healthy adults. Placebo‐ and predose‐adjusted mean differences in QTcF (ddQTcF; primary end point) were analyzed, and solriamfetol pharmacokinetics were characterized. Fifty‐five participants completed all periods. Upper bounds of 2‐sided 90% confidence intervals (CIs) for ddQTcF for both solriamfetol doses were <10 milliseconds at all postdose time points. Assay sensitivity was demonstrated with moxifloxacin; lower bounds of 2‐sided 90%CIs for ddQTcF > 5 milliseconds at 1, 2, and 3 hours postdose. There were no QTcF increases > 60 milliseconds or QTcF values > 480 milliseconds at either solriamfetol dose. Solriamfetol median t_max_ was 2‐3 hours; exposure was dose‐proportional. More participants experienced adverse events (AEs) after solriamfetol 900 versus 300 mg (70% vs 29%); none were serious (all mild/moderate), and there were no deaths. Common AEs were nausea, dizziness, and palpitations. Neither solriamfetol dose resulted in QTcF prolongation > 10 milliseconds.

Solriamfetol is a dopamine and norepinephrine reuptake inhibitor^1^ approved in the United States and European Union to improve wakefulness in adult patients with excessive daytime sleepiness (EDS) associated with narcolepsy (75‐150 mg/day) or obstructive sleep apnea (OSA; 37.5‐150 mg/day).^2^, ^3^, ^4^ In vitro studies have shown that the action of solriamfetol differs from that of traditional stimulants (eg, amphetamines) and other reuptake inhibitors (eg, modafinil).^1^ Solriamfetol exhibits dual activity at dopamine and norepinephrine transporters and lacks monoamine‐releasing effects associated with amphetamines.^1^ Solriamfetol is rapidly absorbed following oral administration, has a terminal half‐life of approximately 6 hours, and is mostly excreted in the urine unchanged (>90%).^5^ Renal impairment increases overall exposure to solriamfetol, with greater increases in exposure as the level of renal function worsens.^6^

The safety and efficacy of solriamfetol (maximum dose evaluated, 300 mg) in reducing EDS in patients with narcolepsy or OSA have been established in phase 3 clinical studies, including 6‐ and 12‐week randomized, double‐blind, placebo‐controlled studies and a 1‐year open‐label extension study.^7^, ^8^, ^9^, ^10^ Solriamfetol has low potential for drug‐drug interactions, including a lack of interaction with oral hormonal contraceptives (a concern with some wake‐promoting agents), and relatively low abuse potential (similar to or lower than that of phentermine).^2^, ^3^, ^11^

In vitro and in vivo nonclinical safety pharmacology studies have indicated minimal effects of solriamfetol on assessments of cardiovascular function. Specifically, solriamfetol at concentrations up to 10 µM (approximately 2.2‐ and 2.6‐fold the maximum plasma concentration [total and unbound] at the highest approved dose of 150 mg/day) did not reduce rapid potassium current in the human ether‐a‐go‐go‐related gene (hERG) assay, had no notable effects on cardiac contractility in isolated guinea pig atria, and had no relevant effects on electrophysiological parameters or early after‐depolarizations in isolated rabbit Purkinje fibers. Notably, solriamfetol did not result in QT interval corrected for heart rate (QTc) prolongation in anesthetized rats, guinea pigs, or dogs or in conscious, unrestrained telemeterized beagles (data on file).

To better characterize the effect of solriamfetol on QT/QTc, a thorough QT/QTc study was conducted. The primary objective of this study was to assess the effect of solriamfetol at doses of 300 and 900 mg (2 and 6 times greater, respectively, than the maximum recommended dose of 150 mg) on QT interval corrected for heart rate (HR) using the Fridericia formula (QTcF) in healthy adult participants.

## Methods

The protocol was approved by the institutional review board (IRB) for the study center (Chesapeake IRB, Columbia, Maryland), and the study was conducted in accordance with standard International Conference on Harmonisation of Technical Requirements for Registration of Pharmaceuticals for Human Use (ICH), U.S. Food and Drug Administration (FDA) regulatory requirements, Good Clinical Practice/ICH, the US Code of Federal Regulations pertaining to clinical studies, and the Declaration of Helsinki. Participants provided written informed consent before enrollment. The study was conducted from September 30, 2015, through October 21, 2015, at a single center in the United States (Celerion, Tempe, Arizona).

### Study Design

This phase 1 randomized, double‐blind, placebo‐ and positive‐controlled, 4‐period crossover study compared the effects of single doses of 300 and 900 mg solriamfetol relative to 400 mg moxifloxacin (positive control) and placebo on QTcF prolongation. Solriamfetol pharmacokinetics (PK) were also characterized.

The selection of solriamfetol doses used in this study (ie, 300 and 900 mg) was based on guidance from the FDA and ICH, which recommend testing at substantial multiples of the anticipated maximum therapeutic exposure, if not precluded by considerations of safety or tolerability.^12^, ^13^ As previously noted, the 300‐mg dose was the highest dose evaluated in phase 3 studies in participants with narcolepsy or OSA.^7^, ^8^, ^9^ The 900‐mg dose was selected for this study because it was likely to be the highest dose that could be studied without having an adverse impact on tolerability or study discontinuation and because it was predicted to result in exposures greater than what would be estimated in individuals who might have increased exposure to therapeutic doses of solriamfetol (eg, those with renal impairment^6^).

### Participants

Eligible participants were healthy men and nonpregnant, nonlactating women (18‐55 years old) weighing at least 52 kg (men) or 45 kg (women) with normal body mass index (19‐30 kg/m^2^). Additional inclusion criteria included nonusers of nicotine‐containing products (≥3 months before first dose until the end of the study) and, for women of childbearing potential, use of a medically accepted method of birth control (≥2 months before first dose until ≥30 days after study completion). Main exclusion criteria included, but were not limited to, a medical history, physical findings, laboratory examination, or electrocardiogram (ECG) findings that might confound the results of the study or pose a risk to the participant; history or the presence of any disease or condition that could interfere with absorption, distribution, metabolism, or excretion of drugs; and participation in a prior study of solriamfetol.

### Protocol

Following a screening period of up to 28 days and confirmation of eligibility, participants were randomly assigned to 1 of 4 treatment sequences that were generated using the Williams method^14^ for Latin square design using a block size of 4. Randomization was stratified by sex. Each participant received each of the 3 active doses (300 and 900 mg solriamfetol and 400 mg moxifloxacin) and placebo on separate days in a complete crossover design in the order that corresponded to their randomly assigned sequence.

During each of the 4 periods, participants checked into the center on the day before dosing (ie, days −1, 7, 14, and 21), clinical and laboratory assessments were performed to ensure eligibility, and participants stayed for 2 days for each period. On the following day (ie, days 1, 8, 15, and 22), data were collected including vital signs, as well as predose ECG data and blood samples for PK analysis. Following the collection of predose data, participants received a blinded dose of study medication; dosing occurred in the morning on each of the 4 dosing days, after an overnight fast of at least 8 hours.

On each dosing day, participants received 3 identical‐looking capsules (each capsule contained placebo or a 300‐mg solriamfetol tablet) and 1 tablet (containing 400 mg moxifloxacin or placebo). Solriamfetol 300‐mg tablets, overencapsulated in opaque gelatin capsules and matching placebo capsules, were provided by the sponsor. Moxifloxacin neat film‐coated tablets (Avelox; Bayer Healthcare Pharmaceuticals Inc., Whippany, New Jersey) and matching placebo tablets were obtained by the pharmacy at the study site. ECG and blood samples were collected for 24 hours postdose. Following a second overnight stay and completion of the 24‐hour sampling time, participants were discharged. A 7‐day washout period between doses occurred after dosing in periods 1, 2, and 3. A safety follow‐up call was conducted approximately 7 ± 2 days after discharge from period 4 (or early study termination).

For the assessment of QT/QTc intervals, Holter monitors (M12R Ambulatory 12‐lead ECG, Global Instrumentation, LLC, Manlius, New York) were used to collect 12‐lead ECGs from approximately 1 hour before dosing through 24 hours after dosing during each of the 4 treatment periods. QT/QTc interval following dosing was assessed from ECG data extracted from the Holter monitor at the following times: 1, 0.75, and 0.5 hours predose (−1, −0.75, and −0.5) and 1, 2, 3, 4, 6, 8, 12, and 24 hours postdose.

At each scheduled time point, three 10‐second extractions were obtained from the Holter monitor within a 5‐minute time window ending at the nominal point. A single reader from the central cardiac laboratory (Celerion, Tempe, Arizona) was used for the review of ECGs from a particular participant. The ECG recordings were measured and classified by software from AMPS, LLC, New York City, New York; all extracted ECG recordings were automatically measured by CalECG.^15^, ^16^, ^17^, ^18^ The quality of the ECG recordings was assessed, and those meeting the quality criteria thresholds were recorded in the database without cardiologist review. All ECG recordings not meeting specific quality criteria thresholds and all waveforms identified for review by the automated algorithm were reviewed by a single board‐certified cardiologist who was blinded to subject, time, and treatment.

Blood samples for PK measurements (4 mL) were collected at the same time points (except for the 1‐ and 0.75‐hour predose times) within a 5‐minute window after the nominal point at predose and 1, 2, 3, 4, 6, 8, 12, and 24 hours postdose. All blood samples were collected in labeled K_2_‐ethylenediaminetetraacetic acid tubes and kept on ice until the samples were centrifuged (within 30 minutes of collection) at ∼3000 revolutions per minute at 4°C for 10 minutes. The plasma was then pipetted into polypropylene tubes for freezing and storage at −20°C until analysis.

Analysis of plasma solriamfetol concentrations were performed by KCAS, LLC, Shawnee, Kansas, using a validated method. Solriamfetol and internal standard, R334898 (JZP‐110‐13C‐d2), were released from the human plasma by protein precipitation and subsequently derivatized using propionic anhydride to form their corresponding propionate products: solriamfetol propionate and R334898 propionate. After derivatization, the products were resolved chromatographically on a reverse phase using a mixture of 0.1% formic acid in water and 0.1% formic acid in methanol as the mobile phase with gradient elution using a 100 × 2.1 mm, 5‐micron particle Betasil C8 analytical column. Solriamfetol and R334898 were detected by monitoring the precursor and product ions (m/z 251.2 →190.2 for derivatized solriamfetol and m/z 254.2 → 193.2 for derivatized R334898) using an Applied Biosystems, Foster City, California, API4000 liquid chromatography‐tandem mass spectrometry unit. The lower limit of quantitation for analysis of solriamfetol in plasma was 8.42 ng/mL, with a range of 8.42‐4210 ng/mL. Inter‐ and intra‐assay relative standard deviation was below 6% for precision and below 2.7% for accuracy. PK blood samples collected during the moxifloxacin and placebo periods were stored. Because PK/pharmacodynamic (PD) modeling was only planned for solriamfetol, moxifloxacin and placebo samples were not analyzed.

Safety and tolerability were assessed throughout the study. Assessments included adverse event (AE) monitoring, vital sign measurements, physical examinations, clinical laboratory tests (chemistry, hematology, and urinalysis), and the Columbia‐Suicide Severity Rating Scale (C‐SSRS). Single 12‐lead safety ECG recordings were obtained at screening, on each check‐in day, before dosing and approximately 2.5 hours after dosing on each dosing day, and on each discharge day and were reviewed by the investigator or qualified designee.

### End Points

The primary PD end points were predose‐adjusted mean difference between solriamfetol (300 and 900 mg) and placebo in QTcF (ddQTcF) and predose‐adjusted mean difference between moxifloxacin (400 mg) and placebo in QTcF (ddQTcF).

Additional PD end points included the number and percentage of participants with any postdose absolute QTcF >450, >480, or >500 milliseconds; number and percentage of participants with predose‐adjusted mean change in QTcF (dQTcF) above a predefined threshold (QTcF increase from predose >30 or >60 milliseconds); number and percentage of participants with any postdose abnormal ECG morphology; mean absolute QTcF, QTcB (QT interval corrected for HR using the Bazett formula), RR, and PR intervals, QRS duration, and derived ventricular HR for each interval; predose‐adjusted mean changes for ECG parameters (dQTcF, dQTcB, dRR, dPR, dQRS, and dHR and for placebo); and placebo‐ and predose‐adjusted mean difference in QTcB (ddQTcB).

In addition, the relationship between placebo and predose‐adjusted mean differences in QTcF (ddQTcF) and plasma concentrations of solriamfetol was assessed.

Solriamfetol PK parameters were calculated by noncompartmental methods using Phoenix WinNonlin version 6.3, and included area under the plasma concentration‐versus‐time curve (AUC) from time 0 to time t of the last quantifiable concentration (AUC_0‐t_), AUC from time 0 to infinity (AUC_0‐inf_), maximum plasma concentration (C_max_), time to peak plasma concentration (t_max_), apparent terminal elimination half‐life (t_1/2_), and apparent oral clearance (CL/F).

Safety variables included treatment‐emergent AEs (TEAEs), vital signs, clinical laboratory results (chemistry, hematology, and urinalysis), 12‐lead ECG findings, and C‐SSRS score.

### Data Analyses

ECG parameters were analyzed using the ECG‐evaluable population, which included participants who had completed the placebo and at least 1 solriamfetol treatment period, had at least 1 evaluable postdose ECG recording for both these treatments, and had no major protocol deviation. Participants who were included in the ECG‐evaluable population but who had missing data for a particular end point were not included in the analysis for that end point. PK and safety data were analyzed using the safety population, which included all participants who received at least 1 dose of study drug.

ECG data included HR and the following intervals: PR, RR, QRS, QT, QTcB, and QTcF. QTcF was the primary and a priori correction method because of the effects of solriamfetol on HR and was calculated as QTcF = QT/(RR)^1/3^.

For all cardiodynamic ECG parameters, the average of the triplicate measurements was rounded to the nearest integer. The predose value for ECG parameters was defined as the mean of the 3 measurements recorded before day 1 dosing in each period (−1, −0.75, and −0.5 hours). The placebo‐ and predose‐adjusted mean difference in QTcF intervals (ddQTcF) were calculated as the predose‐adjusted value for active treatment minus the predose‐adjusted value for placebo at each matching postdose time point.

Analysis of the primary end point, ddQTcF, was performed using a repeated mixed‐effects analysis of covariance (ANCOVA) model that included treatment sequence, treatment period, sex, treatment, time, and time‐by‐treatment interaction as fixed effects and subject nested within sequence as a random effect; 2‐sided 90% confidence intervals (CIs) for ddQTcF were constructed at each point. Upper bounds of the 2‐sided 90%CIs could not exceed 10 milliseconds to conclude that this was a negative study according to ICH E14 guidance.^13^ No type I error adjustment for multiple comparisons across the times was performed. The analyses were performed using SAS PROC MIXED. The model was used to test if dQTcF between treatment was equal to 0 at each time point. For each dose level, the model was also used to estimate the differences in dQTcF least‐squares mean effect between solriamfetol and placebo (ddQTcF) at each time point.

To determine assay sensitivity, the predose‐adjusted mean difference between moxifloxacin and placebo (ddQTcF) was evaluated using time trend plots and 2‐sided 90%CIs at each time point based on the mixed‐effects ANCOVA model. Assay sensitivity was tested at 1, 2, and 3 hours postdose (time points at which maximum QTc prolongation was likely to occur with moxifloxacin). Assay sensitivity was established if lower bounds of the corresponding 2‐sided 90%CI were >5 milliseconds. Hochberg adjustment was used to control for multiplicity at the 0.05 level of significance across comparisons at the 3 time points. With 52 participants who completed all 4 periods per protocol, there was at least 80% power to detect a predose‐adjusted mean difference of 5 milliseconds on the QTc interval between moxifloxacin and placebo. This calculation assumed a common standard deviation of 10 and a 1‐sided significance level of 0.05 using a *t* test. A total of 60 participants were planned in order to obtain data from approximately 52 participants completing all 4 periods per protocol.

PD measures of central tendency were summarized descriptively by treatment, including number and percentage of participants with QTcF intervals ≤450, >450 and ≤480, >480 and ≤500, and >500 milliseconds, and dQTcF intervals (predose adjusted) ≤30 milliseconds (including decrease from predose), >30 and ≤60 milliseconds, and >60 milliseconds.

Correlation analyses were performed to evaluate the relationship between the placebo‐ and predose‐adjusted mean difference in QTcF (ddQTcF) and plasma concentrations of solriamfetol. Pearson correlation coefficients were calculated separately for each treatment.

Solriamfetol plasma concentrations, PK parameters, and safety data were summarized descriptively. For the calculation of PK summary statistics, values that were below the limit of quantification (below 8.42 ng/mL) were treated as 0. AEs were summarized by treatment at onset.

## Results

### Participants

Of 114 potential participants screened, a total of 60 participants entered the study, were randomized, and received study drug; 15 participants were assigned to each of the 4 treatment sequences. All 60 enrolled participants received at least 1 dose of study drug and were included in the safety population. The total ECG‐evaluable population included 59 participants (evaluable data by treatment: placebo, n = 59; solriamfetol 300 mg, n = 56; solriamfetol 900 mg, n = 59; moxifloxacin, n = 58). A total of 57 participants completed the study; 2 participants discontinued because of AEs and 1 discontinued for personal reasons.

Demographics and baseline characteristics were generally similar across treatment groups (Table 1). The majority of participants were white (92%) and of Hispanic or Latino ethnicity (77%), there were more women than men (57% vs 43%), and the mean ± SD age was 36.9 ± 8.98 years.

**Table 1:** Demographics and Baseline Characteristics of Participants (Safety Population)

	Treatment Sequence				
Demographic Characteristics	ABDC, n = 15	BCAD, n = 15	CDBA, n = 15	DACB, n = 15	Overall, N = 60
Sex, n (%)					
Female	9 (60)	8 (53)	9 (60)	8 (53)	34 (57)
Male	6 (40)	7 (47)	6 (40)	7 (47)	26 (43)
Age (years), mean (SD)	37.8 (8.0)	33.3 (8.8)	39.1 (7.1)	37.5 (11.4)	36.9 (9.0)
Weight (kg), mean (SD)	70.5 (10.4)	69.6 (13.0)	69.4 (9.6)	66.4 (7.3)	69.0 (10.2)
BMI (kg/m^2^), mean (SD)	26.6 (1.9)	25.2 (2.3)	26.1 (2.6)	24.1 (2.6)	25.5 (2.5)
Race, n (%)					
Black or African American	0 (0)	2 (13)	2 (13)	0 (0)	4 (7)
Native Hawaiian or other Pacific Islander	0 (0)	0 (0)	0 (0)	1 (7)	1 (2)
White	15 (100)	13 (87)	13 (87)	14 (93)	55 (92)
Ethnicity, n (%)					
Hispanic or Latino	14 (93)	11 (73)	12 (80)	9 (60)	46 (77)
Not Hispanic or Latino	1 (7)	4 (27)	3 (20)	6 (40)	14 (23)

BMI, body mass index; SD, standard deviation.

Treatment A: 300 mg solriamfetol; treatment B: 900 mg solriamfetol; treatment C: 400 mg moxifloxacin (positive control); treatment D: placebo.

### PD Analyses

**Figure 1:**
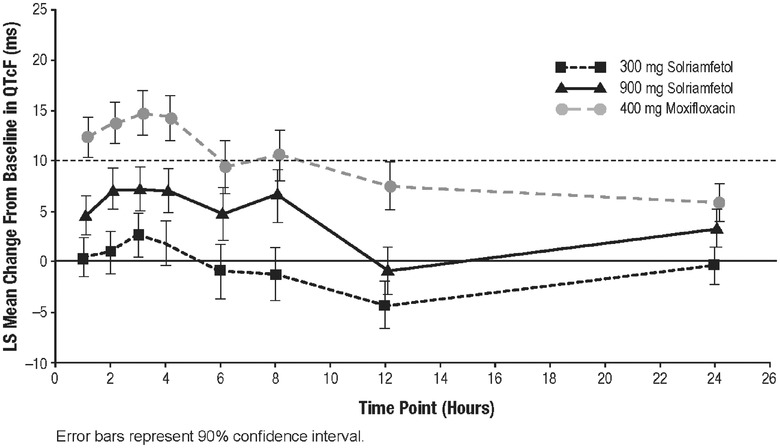
Placebo‐ and predose‐adjusted mean differences in QTcF (ddQTcF) versus time (ECG‐evaluable population). ECG, electrocardiogram; LS, least squares; QTcF, QT interval with Fridericia correction.

The mixed‐effects ANCOVA model analysis showed that the lower bounds of the 2‐sided 90%CIs for the difference in mean dQTcF between moxifloxacin and placebo (ddQTcF) were all >5 milliseconds at 1, 2, and 3 hours after dosing (Figure 1); therefore, assay sensitivity was established.

For the primary PD end point (ddQTcF), the upper bounds of the 2‐sided 90%CI for the mean difference between the 300‐ or 900‐mg solriamfetol doses and placebo were <10 milliseconds at all postdose time points (Figure 1). Therefore, this was a negative thorough QT study of solriamfetol (300 or 900 mg) according to ICH E14 guidance.

Categorical analysis of QTcF intervals showed that no participants had an increase in QTcF > 60 milliseconds from baseline following any treatment; an increase from baseline > 30 milliseconds was observed for 4 participants following 900 mg solriamfetol and for 2 participants following moxifloxacin administration (Table 2). Maximum postdose QTcF intervals did not exceed 480 milliseconds following any treatment; maximum postdose QTcF > 450 milliseconds was observed for 1 participant each following placebo and solriamfetol 300 mg, 2 participants following 900 mg solriamfetol, and 8 participants following moxifloxacin administration.

**Table 2:** Categorical Analysis of QTcF (ECG‐Evaluable Population)

QTcF (ms), n (%)	Placebo, n = 59	Solriamfetol 300 mg, n = 56	Solriamfetol 900 mg, n = 59	Moxifloxacin 400 mg, n = 58
Observed value				
≤450	58 (98)	55 (98)	57 (97)	50 (86)
>450 to ≤480	1 (2)	1 (2)	2 (3)	8 (14)
>480 to ≤500	0	0	0	0
>500	0	0	0	0
Change from baseline				
≤30	59 (100)	56 (100)	55 (93)	56 (97)
>30 to ≤60	0	0	4 (7)	2 (3)
>60	0	0	0	0

ECG, electrocardiogram; QTcF, QT interval corrected for heart rate using the Fridericia formula.

**Figure 2:**
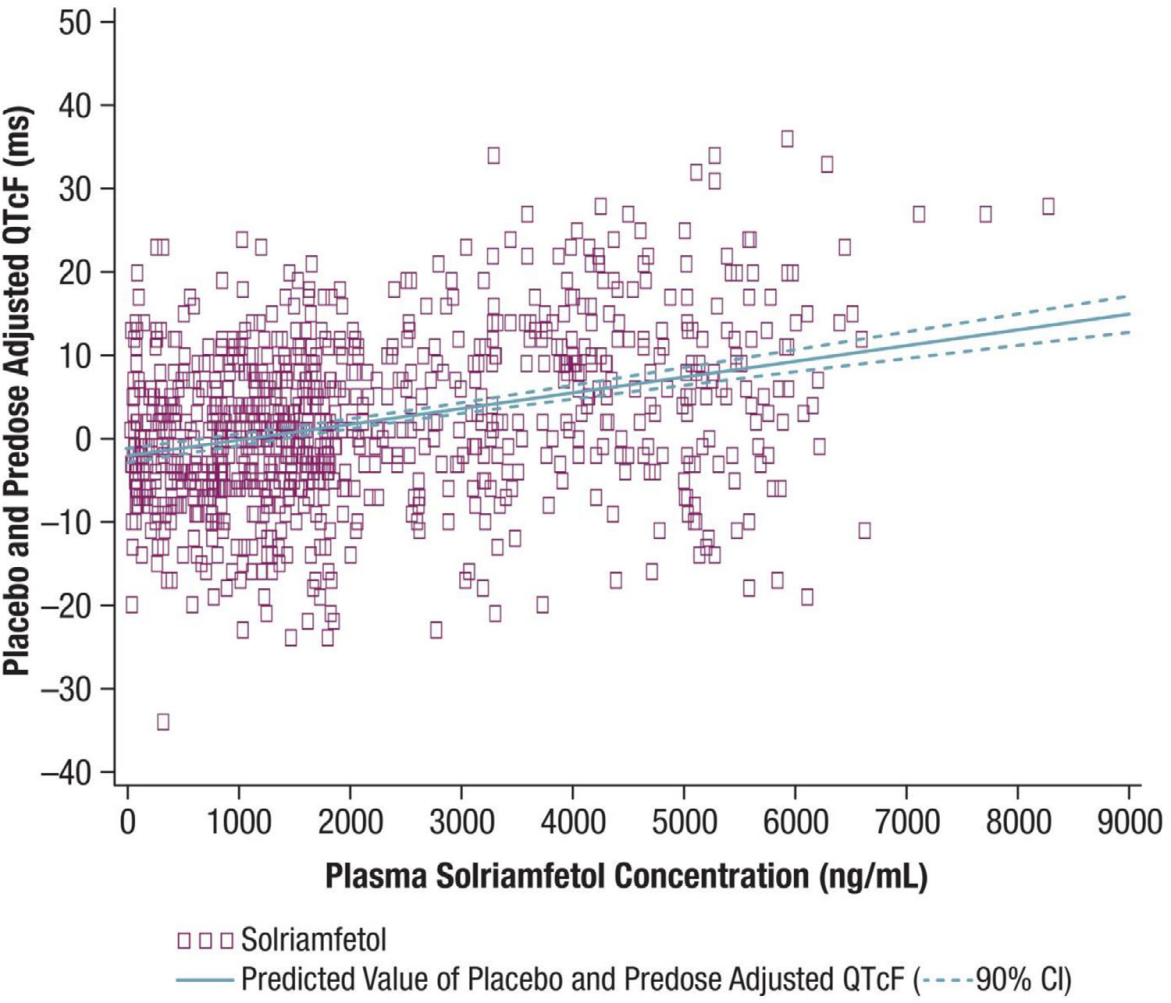
Placebo‐ and predose‐adjusted mean differences in QTcF (ddQTcF), in milliseconds, versus solriamfetol concentrations (ng/mL) following administration of solriamfetol 300 and 900 mg (ECG‐evaluable population). ddQTcF = intercept + slope x concentration. Slope, 0.00189 (90%CI, 0.00158 to 0.0022); intercept, –1.96753 (90%CI, −2.83147 to −1.10358); *R* ^2^ = 0.1038. 90%CI is based on mean predicted values. *P* (slope) < 0.0001. CI, confidence interval; ECG; electrocardiogram; QTcF, QT interval with Fridericia correction.

Results of the ddQTcF‐concentration correlation analysis showed that the mean slope of the regression equation was 0.00189 milliseconds per ng/mL (90%CI, 0.00158‐0.0022 milliseconds per ng/mL; *R* ^2^ = 0.1038; Figure 2). At a C_max_ of 1774 ng/mL, following the 300‐mg dose, the estimated mean ddQTcF (upper 90% confidence bound) was 1.3843 (1.9338). At a C_max_ of 5290 ng/mL, following the 900‐mg dose, the estimated mean ddQTcF (upper 90% confidence bound) was 8.0274 (9.1173).

### PK Analyses

**Figure 3:**
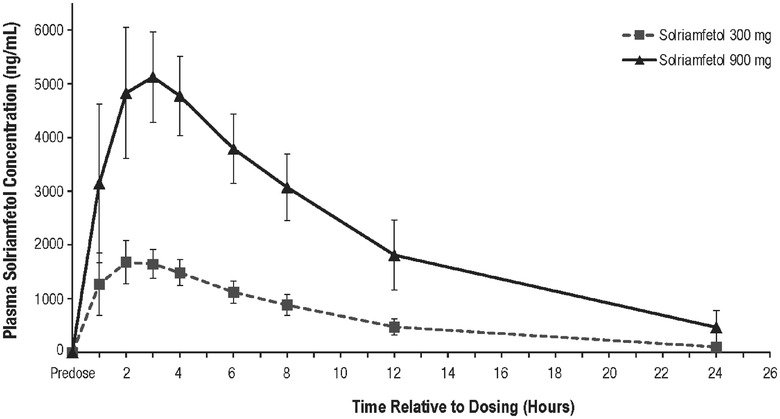
Mean (SD) solriamfetol plasma concentrations over time profiles following 300 and 900 mg administrations (safety population, n = 56). SD, standard deviation.

Solriamfetol was rapidly absorbed, with median t_max_ ranging from 2 to 3 hours following oral administration of the 300‐ and 900‐mg doses (Figure 3). As presented in Table 3, the mean C_max_ was approximately 3‐fold higher (5290 and 1774 ng/mL, respectively), and the mean AUC_0‐inf_ was approximately 3.5‐fold greater (59 910 and 16 970 ng·h/mL, respectively) following the 900‐mg dose compared with the 300‐mg dose, indicating dose proportionality; mean t_1/2_ was similar for both doses (5.09 and 5.79 hours for 300 and 900 mg, respectively). Mean CL/F values were similar at the 2 doses (18.45 and 16.22 L/h following the 300‐ and 900‐mg doses, respectively).

**Table 3:** Solriamfetol Pharmacokinetic Parameters (Safety Population)

Parameter (Units)	Solriamfetol 300 mg, n = 56^a^	Solriamfetol 900 mg, n = 56^b^
C_max_ (ng/mL), mean (SD)	1774 (342.5)	5290 (908.6)
t_max_ (h), median (range)	2.0 (1.0‐4.1)	3.0 (1.0‐4.1)
t_1/2_ (h), mean (SD)	5.1 (1.3)	5.8 (1.6)
AUC_0‐t_ (ng·h/mL), mean (SD)	16 120 (3101.7)	54 600 (12 169)
AUC_0‐inf_ (ng·h/mL), mean (SD)	16 970 (3563.1)	59190 (15 788)
CL/F (L/h), mean (SD)	18.5 (3.9)	16.2 (4.1)

AUC_0‐inf_, area under concentration‐time curve from zero to infinity; AUC_0‐t_, area under concentration‐time curve from zero to time t; CL/F, apparent oral clearance; C_max_, maximum plasma concentration; SD, standard deviation; t_1/2_, terminal elimination half‐life; t_max_, time to reach maximum plasma concentration.

^a^ Four participants did not receive the 300‐mg dose and, therefore, were not included in the PK descriptive statistics.

^b^ Four participants vomited within 2 times the median t_max_ following a single dose of 900 mg solriamfetol; therefore, they were excluded from the PK descriptive statistics.

### Safety/Tolerability

TEAEs were reported by 46 (77%) of the 60 participants (Table 4). There were more TEAEs following solriamfetol 900 mg versus 300 mg or placebo (70%, 29%, and 12%, respectively). The most frequently reported types of TEAEs included nervous system disorders (67%), gastrointestinal disorders (50%), and psychiatric disorders (47%). The most frequent AEs occurring in ≥5% of participants on solriamfetol 300 mg included nausea, dizziness, palpitations, and headache. The most frequent AEs occurring in ≥25% of participants on solriamfetol 900 mg included nausea, dizziness, and palpitations. No seizures, cardiac dysrhythmia, or syncope were reported. All AEs were mild or moderate in severity, and no serious AEs or deaths were reported.

**Table 4:** Most Frequently Reported TEAEs (Safety Population)^a^

Preferred Term, n (%)	Placebo, n = 59	Solriamfetol 300 mg, n = 56	Solriamfetol 900 mg, n = 60	Moxifloxacin 400 mg, n = 58	Total, N = 60
Any adverse event	7 (12)	16 (29)	42 (70)	11 (19)	46 (77)
Nausea	0 (0)	4 (7)	20 (33)	6 (10)	26 (43)
Dizziness	1 (2)	4 (7)	18 (30)	4 (7)	20 (33)
Headache	2 (3)	4 (7)	14 (23)	4 (7)	17 (28)
Palpitations	0 (0)	3 (5)	17 (28)	0 (0)	19 (32)
Anxiety	0 (0)	2 (4)	12 (20)	1 (2)	14 (23)
Insomnia	0 (0)	2 (4)	8 (13)	2 (3)	12 (20)
Asthenia	0 (0)	2 (4)	7 (12)	1 (2)	9 (15)
Chest discomfort	0 (0)	2 (4)	7 (12)	1 (2)	8 (13)
Paresthesia	0 (0)	1 (2)	12 (20)	0 (0)	13 (22)
Nervousness	0 (0)	1 (2)	9 (15)	0 (0)	10 (17)
Dizziness, postural	0 (0)	0 (0)	13 (22)	2 (3)	13 (22)
Dry mouth	0 (0)	0 (0)	12 (20)	1 (2)	12 (20)
Vomiting	0 (0)	0 (0)	8 (13)	0 (0)	8 (13)
Dyspnea	0 (0)	0 (0)	7 (12)	1 (2)	8 (13)
Tremor	0 (0)	0 (0)	6 (10)	2 (3)	8 (13)
Feeling hot	0 (0)	0 (0)	6 (10)	0 (0)	6 (10)

TEAEs, treatment‐emergent adverse events.

^a^ Events occurring in ≥10% of participants.

Two participants discontinued the study because of AEs. One participant received solriamfetol 300 and 900 mg in periods 1 and 2, respectively, and discontinued because of elevated alanine aminotransferase following administration of placebo in period 3. One participant who received moxifloxacin and placebo in periods 1 and 2, respectively, discontinued because of mild tardive dyskinesia following administration of solriamfetol 900 mg in period 3.

No clinically significant ECG abnormalities were found in any treatment group. Transient, dose‐dependent increases in HR were observed, and the mean HR remained within normal range. Blood pressure (BP) increase from baseline at discharge (∼24 hours postdose) was observed after dosing with solriamfetol 900 mg (median changes in systolic blood pressure [SBP] and diastolic blood pressure [DBP] were ∼5 mm Hg), and a minimal increase was noted for solriamfetol 300 mg. Mean BP remained within normal range.

## Discussion

In this study, neither dose (300 or 900 mg) of solriamfetol resulted in QTcF prolongation, as defined by the upper bound of a 2‐sided 90%CI for ddQTcF exceeding 10 milliseconds. In contrast, the lower bound of the 2‐sided 90%CI for QTcF following administration of moxifloxacin 400 mg was >5 milliseconds, establishing assay sensitivity for the study. Thus, the study met the criteria for a negative thorough QT study per ICH E14 guidance.^13^

Results from QTcF categorical analyses were supportive of the ddQTcF end point. Specifically, there were no increases from baseline QTcF > 60 milliseconds nor any absolute QTcF values > 480 milliseconds in any participants at either dose of solriamfetol. Furthermore, no participant had an increase from baseline QTcF > 30 milliseconds following administration of 300 mg solriamfetol. In the correlation analysis of ddQTcF and plasma solriamfetol concentrations, the upper bounds of the 90%CI for ddQTcF were <10 milliseconds at the mean C_max_ corresponding to either solriamfetol dose; thus, these results were consistent with the ddQTcF analysis.

The QTcF findings in this study are consistent with previous phase 2 and 3 clinical studies, which included participants with narcolepsy or OSA, who may be more susceptible to arrhythmogenic effects because of their underlying disease compared with healthy populations. Although these studies did not systematically evaluate QTcF as an outcome as was done in the current study, clinical assessments were conducted based on vital signs, ECG, and reported AEs.^7^ At doses up to 300 mg, solriamfetol has been shown to cause small changes from baseline in SBP (−0.5 to 2.8 mm Hg), DBP (−0.1 to 3.0 mm Hg), and pulse rate (0.2 to 4.8 beats per minute) in studies in patients with narcolepsy or OSA^7^, ^8^, ^9^; however, no clinically significant ventricular arrhythmias including torsades de pointes (TdP) or morphology changes on ECG have been reported.^5^, ^6^, ^7^ Collectively, these data suggest a lack of clinically meaningful effects of solriamfetol on ECG parameters, and the totality of the available evidence does not suggest cause for concern about QTc prolongation or TdP with solriamfetol.

Published QTc data for other wake‐promoting treatments are limited. Pooled data from 6 randomized, placebo‐controlled trials suggest that clinically meaningful ECG changes with modafinil are similar to those with placebo and suggest a lack of treatment‐emergent ECG abnormalities.^19^ In a 12‐month study of armodafinil (n = 323), reported ECG changes included QTcF >450 milliseconds (n = 13) or >500 milliseconds (n = 1), QTcF change from baseline > 60 milliseconds (n = 3), and AEs of long QT syndrome (n = 1) and QRS complex prolonged (n = 1).^20^ Pitolisant prolongs the QT interval, and it is recommended to avoid use in patients with known QT prolongation, in combination with other drugs known to prolong QT interval and in patients with a history of cardiac arrhythmias.^3^

The PK profile of solriamfetol in this study is consistent with what has previously been reported in healthy adults.^5^, ^6^ Solriamfetol was rapidly absorbed, with a median t_max_ of 2 and 3 hours following oral administration of 300‐ and 900‐mg doses, respectively.

An approximately 3‐fold higher solriamfetol C_max_ and 3.5‐fold greater AUC_0‐inf_ were observed following the 900‐mg dose compared with the 300‐mg dose, indicating dose‐proportional PK. The mean half‐life of 5 to 6 hours was similar for the 300‐ and 900‐mg doses.

The tolerability of solriamfetol at the 300‐mg dose was consistent with what has been observed in clinical efficacy studies.^7^, ^8^, ^9^ All TEAEs in this study were mild or moderate in severity, with nausea, dizziness, palpitations, and headache (solriamfetol 300‐mg dose only) reported most frequently. As expected, the higher dose of 900 mg solriamfetol was associated with more TEAEs than the 300‐mg dose. Specific AEs that were more frequent following the 900‐mg dose compared with the 300‐mg dose or placebo were nausea, dizziness, palpitations, headache, dizziness postural, paresthesia, dry mouth, and anxiety.

## Conclusions

This thorough QT/QTc study demonstrated that single doses of solriamfetol at doses of 300 and 900 mg met the criteria for a negative thorough QT study per ICH E14 guidance. The PK and safety/tolerability profiles were consistent with what has been previously reported.

## Conflicts of Interest

K. Zomorodi, D. Chen, and L. Carter are employees of Jazz Pharmaceuticals, who in the course of their employment, have received stock options exercisable for, and other stock awards of, ordinary shares of Jazz Pharmaceuticals plc. L . Lee is a former employee of Jazz Pharmaceuticals, who in the course of his employment, has received stock options exercisable for, and other stock awards of, ordinary shares of Jazz Pharmaceuticals plc. D. Swearingen has nothing to disclose.

## Funding

This study was supported by Jazz Pharmaceuticals. Jazz Pharmaceuticals has worldwide development, manufacturing, and commercialization rights to solriamfetol, excluding certain jurisdictions in Asia. SK Biopharmaceuticals, the discoverer of the compound (also known as SKL‐N05), maintains rights in 12 Asian markets, including Korea, China, and Japan.

## Author Contributions

Study concept and design: Carter, Chen, Zomorodi.

Acquisition of data: Swearingen, Zomorodi.

Study investigator: Swearingen.

Data analysis and interpretation: Carter, Chen, Zomorodi.

Enrolled patients: Swearingen.

Drafting of the manuscript: all authors.

Critical revision of the article for important intellectual content: all authors.

## Professional Medical Writing Disclosure

Under the direction of the authors, Sherri Jones, PharmD, and Jeannette Fee of Peloton Advantage, LLC, an OPEN Health company, provided medical writing and editorial support for this article, which was funded by Jazz Pharmaceuticals.

## Data‐Sharing Statement

All relevant data are provided within the article and supporting files.
